# Treatment of dysphagia after stroke with acupuncture and related therapies: A protocol for systematic review and network meta-analysis

**DOI:** 10.1097/MD.0000000000021657

**Published:** 2020-08-21

**Authors:** Fanjie Xiong, Kai Song, Ailing Huang, Hong Zhang

**Affiliations:** College of acupuncture and Tuina, Chengdu university of Traditional Chinese Medicine, Chengdu, Sichuan, China.

**Keywords:** dysphagia, stroke, acupuncture and related therapies, protocol, systematic review, network meta-analysis

## Abstract

**Background:**

Dysphagia is a common complication after stroke, with high disability rate and high fatality rate. Although several clinical studies and evidence-based medicine have demonstrated the efficacy of acupuncture in the treatment of dysphagia after stroke, there are significant differences in study design and intervention methods. The objective of this study is to compare the efficacy and safety of different acupuncture and related therapies in the treatment of dysphagia after stroke, so as to provide a superior clinical program.

**Methods:**

We will search 7 databases for randomized controlled trials of acupuncture-related therapies for dysphagia after stroke, including PubMed, the Cochrane Library, EMbase, China National Knowledge Infrastructure, China Biological Medicine, Chinese Scientific Journals Database, and wan-fang databases, from the date of the establishment of each database to March 31, 2020. The network meta-analysis will be implemented through Aggregate Data Drug Information System 1.16.8 and Stata 13.0 software. Clinical Efficiency, videofluoroscopic swallowing study score and Kubota Drinking Water Test grade will be the primary outcomes, Swallowing disorder specific quality of life score, Standardized Assessment and Adverse effects will be evaluated as secondary outcomes. Mean differences or odds ratios will be used for statistical analysis. We will ensure the reliability of the results through node-split model and heterogeneity analysis. In addition, methodological quality will be evaluated based on the Cochrane Collaboration's tool, and the quality of evidence will be evaluated according to the Grading of Recommendations Assessment, Development and Evaluation system.

**Results:**

This study will provide a reliable evidence for the selection of acupuncture and related therapies for dysphagia after stroke.

**Conclusion:**

The results of this study will provide references for evaluating the influence of acupuncture and related therapies for dysphagia after stroke, and provide decision-making references for clinical research.

**Ethics and dissemination:**

This study did not require ethical approval. We will disseminate our findings by publishing results in a peer-reviewed journal.

**OSF registration number:**

DOI 10.17605/OSF.IO/TAHND.

## 1 Introduction

Dysphagia is 1 of the most common consequences of stroke, which occurs in 27% to 64% of stroke patients,^[1–3]^ and especially in acute post-stroke survivors, with the incidence reached approximately 50%.^[4]^ Dysphagia can improve spontaneously by 2 weeks after the stroke, however, 15% of patients will still have swallowing dysfunction at 1 month ^[5,6]^ Dysphagia after stroke has many complications, including malnutrition, dehydration, and aspiration pneumonia,^[7,8]^ that are related to higher rates of death and disability, and also affect the quality of life of patients.^[9]^ Furthermore, research shows that dysphagia is associated with social anxiety, substance abuse, and depression.^[10]^

However, until today, dysphagia after stroke is an underdiagnosed and undertreated condition around the world, and most patients do not receive comprehensive care.^[11]^ Current treatment for dysphagia in post-stroke includes swallowing training, speech and language therapy, dietary modification, and behavioral interventions.^[12]^ In recent years, there have been a number of emerging therapies for swallowing problem among post-stroke patients, for example, repetitive transcranial magnetic stimulation and transcranial direct current stimulation, transcutaneous and intrapharyngeal electrical stimulation, and non-invasive brain stimulation strategies.^[11]^ However, these interventions are reported to have only a temporary and relatively limited effectiveness.^[12]^ Therefore, a multidisciplinary approach is urgently needed to manage patients with dysphagia after stroke.

Acupuncture, belongs to traditional Chinese medicine practice, which means acupuncurist will insert needles into a person's skin, with the aim of balancing their energy named “qi”. Acupuncture is used routinely in stroke rehabilitation in China and elsewhere in East Asia over the past 1,000 years, and has been recommended by the World Health Organization as an alternative and complementary strategy for stroke treatment and care.^[13,14]^ There are various acupuncture and related therapies for treatment of dysphagia after stroke, including the traditional manual acupuncture (TA), electro-acupuncture (EA), moxibustion acupuncture (MA), and scalp acupuncture (SA), and so on^[15]^ A series of clinical trials and animal studies have shown that the mechanism of acupuncture for stroke mainly includes:

(1) regulation of the release of neurotransmitters;
(2) regulation of cerebral microcirculation;
(3) antiapoptosis;
(4) stimulation of neurogenesis and cell proliferation;
(5) regulation of neuroplasticity.^[16,17]^

Many studies have indicated that acupuncture might be beneficial in the rehabilitation of patients with swallowing dysfunction in post-stroke.^[18–20]^

Several systematic reviews reported that acupuncture and related therapies might be effective for dysphagia after stroke.^[12,19–22]^ However, there has been no network meta-analysis (NMA) of the differences between different acupuncture and related therapies for dysphagia after stroke. The objective of this study is to assess the efficacy and safety of different acupuncture and related therapies, to synthesize all this evidence and perform a comprehensive rank of available acupuncture and related therapies for dysphagia after stroke, and to provide decision-making reference for clinical practitioners, patients, and health policy makers.

## 2 Methods

### 2.1 Protocol and registration

The NMA protocol has been registered on the Open Science Framework (OSF) platform (https://osf.io/tahnd/), registration number: DOI 10.17605/OSF.IO/TAHND. This protocol was drafted and reported in accordance with the Preferred Reporting Items for Systematic Reviews and Meta-Analyses Protocols (PRISMA-P) guidelines.^[23]^ The final report will comply with the recommendations of the PRISMA Extension Statement for Reporting of Systematic Reviews Incorporating Network Meta-analyses of Healthcare Interventions.^[24]^

### 2.2 Ethics

Since NMA does not involve the collection of private information, this research does not require ethical approval.

### 2.3 Eligibility criteria

The participant (P), intervention (I), comparator (C), outcome (O), and study design (S) are the 5 main factors determining the inclusion and exclusion criteria of this research.

#### 2.3.1 Type of study design

This study is a systematic review with NMA of randomized controlled trials (RCTs) on acupuncture and related therapies for dysphagia after stroke. All relevant RCTs using acupuncture therapies for dysphagia after stroke will be included. Quasi-RCTs, duplications, animal trails, review documents, clinical experience, and case reports will be excluded. Additionally, only English and Chinese literature will be searched for this study.

#### 2.3.2 Type of participant

All cases included in the trial will be patients with dysphagia after stroke, regardless of gender, age, educational background, nationality, or outpatient therapy or inpatient therapy. Specific diagnostic criteria include:

(1) ischemic or hemorrhagic stroke confirmed by CT or MRI scan;
(2) dysphagia confirmed by videofluoroscopic swallowing study (VFSS) and/or Mann Assessment of Swallowing Ability.^[25]^

Swallowing disorders caused by other underlying neurological disorders will be excluded.

#### 2.3.3 Type of interventions and comparators

We will consider acupuncture and related therapies (acupoint-based therapy), regardless of needling techniques and stimulation method including TA, EA, MA, SA, moxibustion, catgut embedding, transcutaneous electrical acupoint stimulation, acupoint injection, medium-frequency electric stimulation, and so on. These interventions can be used in any combinations or in combination with conventional therapy (conventional treatment or rehabilitation training in neurology department). Non-acupuncture-related treatments will be excluded, such as medicine, and transcranial electric/magnetic stimulation, and so on.

In order to ensure the consistency and transmissibility of the studies, controlled interventions will be limited to different acupuncture and related therapies, placebo group, or conventional therapy group. Trials comparing 2 acupoint selections or acupuncture manipulations will be excluded.

#### 2.3.4 Type of outcomes

(1) Primary outcomes
  Clinical efficiency (Total effective rate), will be measured as the proportion of effective patients to the total sample size in the studies, after treatment and/or during follow-up. The total effective rate is the sum of the data contained in the original literature on “recovery”, “significant effect”, “effective” and related terms.
  VFSS score, will be measured as the dynamic passage of food from the mouth to the throat and the occurrence of aspiration, leakage, cough and food retention by swallowing angiography before and after treatment.
  Kubota drinking water test grade, will be measured as the time taken to drinking water and the occurrence of choking cough before and after treatment.
(2) Secondary outcomes
  Swallowing disorder specific quality of life score.
  standardized swallowing assessment.
  Adverse effects reported in the original studies (eg, skin or tissue damage, pain or discomfort, vascular, visceral or nerve injury).

The definitions of the outcomes are described in Table 1. Those outcome measures, including clinical efficiency, VFSS score, Kubota Drinking Water Test grade, Swallowing disorder specific quality of life score, and standardized assessment will be used to assess for effectiveness, and the safety assessment will be measured by adverse effects.

**Table 1:**
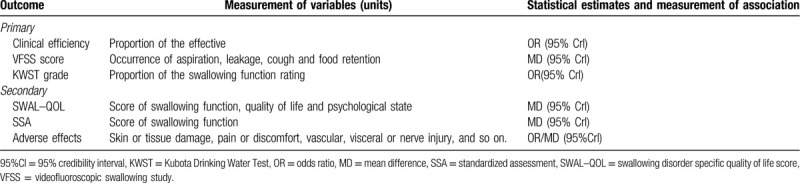
Characteristics of the outcome measures.

### 2.4 Literature retrieval strategy

Computer retrieval of published RCTs of acupuncture and related therapies for dysphagia after stroke is conducted in PubMed, the Cochrane Library (issue 3, 2020), EMbase, China National Knowledge Infrastructure, China Biological Medicine, Chinese Scientific Journals Database (VIP), and wan-fang databases. The time limit of document retrieval is from the establishment of each database to March 31, 2020. The language is limited to English and Chinese. In addition, the World Health Organization International Clinical Trials Registration Platform and Clinical Trials.gov will also be searched for ongoing experiments and Chinese RCTs related to the disease. The used retrieval mode will be a combination of free words and medical subject headings terms, including: “dysphagia”, “stroke”, “acupuncture”, “moxibustion”, “electro-acupuncture”, “moxibustion acupuncture”, “acupoint”, “auricular therapy”, “scalp acupuncture”, etc. The following terms will be used in the Chinese database retrieval: “tunyanzhangai”, “cuzhong”, “zhongfeng”, “zhenjiu”, “aijiu”, “dianzhen”, “wenzhen”, “touzhen”, “xuewei”, “suijiduizhao”, etc. Taking PubMed as an example, the initial search strategy is shown in Table 2 and will be adjusted according to the specific database.

**Table 2:**
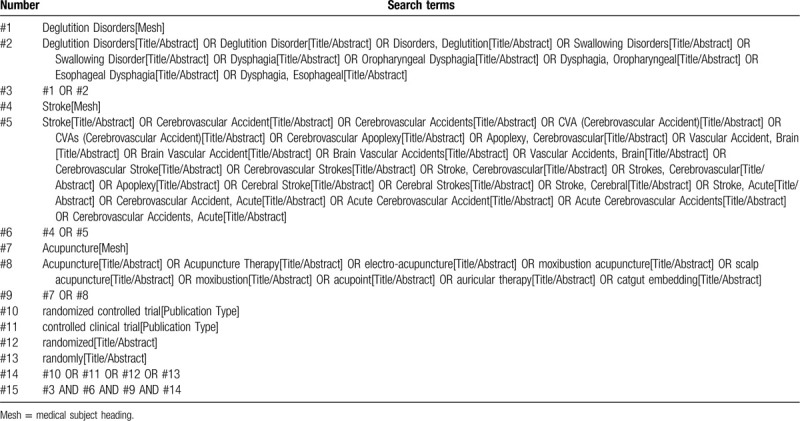
Search strategy of the PubMed.

### 2.5 Literature selection and data extraction

**Figure 1:**
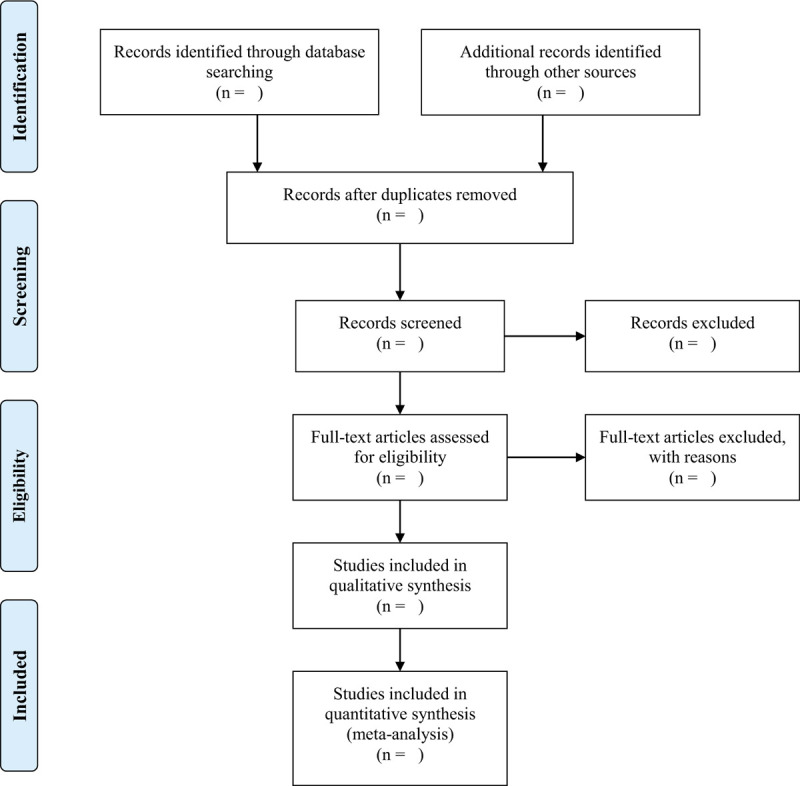
PRISMA flow diagram of the study selection process.

As shown in Figure 1, Two researchers (Fanjie Xiong and Kai Song) will independently screen literatures according to the inclusion and exclusion criteria:

(1) The retrieved literatures will be imported into Endnote X9 software for rechecking, and duplicate references are removed;
(2) By reading the title and preliminaryly screening the abstract, exclude the literature that obviously does not meet the inclusion criteria;
(3) Download and read the full text for re-screening;
(4) After the final inclusion, the pre-designed data extraction table is used for data extraction, and the results will be cross-checked;
(5) If there is any disagreement, the third researcher (Ailing Huang) will be asked to assist in the judgment.

The main content of data extraction includes: basic information of literature (title, journal, author, and publication date), basic situation of the research object (sample size, gender, mean age, intervention and comparator, and course of treatment), and outcome data (numbers of response events, non-response events, dropouts, time points, mean, SD, follow-up time and adverse events). If the required data is lost or incomplete, we will contact the corresponding author of the original document or the relevant email address of the first author. If there is no response, the record is excluded. At the same time, the key factors of bias risk assessment will be extracted.

### 2.6 Risk of bias assessment

The methodological quality of systematic review reflects the risk of bias or validity in its process and results. Methodological quality will be assessed based on the Cochrane Handbook 5.2.0.^[26]^ Two trained researchers (Fanjie Xiong and Kai Song) will independently evaluate the risk of bias of the included studies. In case of dispute, submit to the corresponding author (Hong Zhang) for arbitration. Cochrane bias risk assessment tool will be used to assess the risk of RCTs included in NMA, including:

(1) random sequence generation;
(2) allocation concealment;
(3) blinding of the subjects and researchers;
(4) blinding of outcome assessment;
(5) incomplete outcome data;
(6) selective reporting;
(7) other bias.

### 2.7 Data synthesis and statistical methods

#### 2.7.1 NMA

This study will use Aggregate Data Drug Information System 1.16.8 for NMA.^[27]^ Based on bayesian framework, the software uses Markov Chain-monte Carlo algorithm to prior evaluate and process the extracted data, which provides support for further research and decision making. Preset model parameters: 4 chains will be used for simulation analysis, with an initial value of 2.5, a step size of 10, 20,000 annealing times, and 50,000 simulation iterations. Mean differences or odds ratios will be used as the effect sizes for statistical analysis, both with 95% credible intervals. Firstly, the network evidence plot is generated according to different outcome indicators. The Network evidence plot consists of boxes and lines. The box represents each intervention included in the analysis, while the line represents RCT evidence for a direct comparison between the 2 interventions, and the number represents the number of studies for the direct comparison. According to the results of the NMA, rank probability plot of various modeling methods is generated and sorted by dominance, with Rank top1 being the optimal sequence. However, if the lower score is better in the score standard of the outcome, the lowest ranked prediction sequence is the optimal sequence.

#### 2.7.2 Statistical model selection

Node-split model will be used to verify the consistency of the corresponding data. If there is no statistical difference (*P* > .05) between direct comparison and indirect comparison, the consistency model is used, whereas the inconsistency model will be used for analysis. If the consistency model is adopted, then the stability of the results is verified by the inconsistency model: when the inconsistency factors including 0, and at the same time inconsistency standard deviation including 1 says the result of consistency model is more stable and reliable. At the same time, various analysis models are iterated with preset parameters, and the convergence of iteration effect will be judged by potential scale reduced factor (PSRF). When the PSRF value is close to or equal to 1 (1≤PSRF≤1.05), it indicates that the convergence is complete, the model has good stability, and the conclusion of analysis is reliable. If the PSRF value is not in this range, the iteration continues manually until the PSRF value reaches the range standard.

#### 2.7.3 Assessment of heterogeneity

Before the combination of effect size, the heterogeneity of the included literature is tested using Stata 13.0. When inter-study heterogeneity exists, the random effect model will be used. For comparison of each pair, heterogeneity is assessed by the statistic *I*^*2*^ value. When *I*^*2*^ > 50%, inter-study heterogeneity is considered to be small or there is no obvious heterogeneity. When *I*^*2*^ > 50%, it indicates that there is heterogeneity between studies, and the source of heterogeneity should be further searched. Meta-analysis will be performed after removal of studies where main or unacceptable sources of heterogeneity were derived. Furthermore, if the source of heterogeneity cannot be explored, a narrative review will be provided.

#### 2.7.4 Sensitivity analysis and subgroup analysis

If necessary, the sensitivity analysis will be used to assess the effect of each study on the random effects model. The sensitivity of the general combined effect of all outcome indicators is analyzed by the exclusion method. That is, all studies are excluded 1 by 1, and the remaining studies will be re-analyzed to determine the stability of the results. If there is no qualitative change in the combined effect showed in the results, the results are stable.

Based on the overall situation of the included study, subgroup analysis will be conducted as far as possible:

(1) short-term and long-term efficacy will be discussed according to the treatment and follow-up period;
(2) Average Age of patients;
(3) Intervention time of treatment measures;
(4) Different acupuncture manipulations and acupoint selection.

#### 2.7.5 publication bias

If 10 or more studies are included in the NMA, a comparison-adjusted funnel plot is developed using Stata to evaluate the presence of small sample effects or publication bias in the intervention network. Descriptive analysis will be carried out through the symmetry of funnel plot. If the plot is asymmetric and there is no inverted funnel shape, it indicates that there may be publication bias. This may be related to the difficulty in the publication of the literature with negative results and the low quality of the inclusion methods.

#### 2.7.6 Evaluating the quality of the evidence

To grade evidence quality and understand the current situation of evidence rating thereby analyzing possible problems, the Grading of Recommendations Assessment, Development and Evaluation system will be used to assess the quality of evidence in the NMA ^[28]^ Based on the risk of bias, inconsistency, imprecision, indirection, and publication bias, Grading of Recommendations Assessment, Development and Evaluation grades evidence quality into 4 levels: high, medium, low, and very low.

#### 2.7.7 Patient and public involvement

There was no patient or public involvement in the preparation of this protocol.

## 3 Discussions

There are a significant number of patients with dysphagia after stroke in China, and acupuncture and related therapies have been a common intervention for swallowing dysfunction in post-stroke.^[29]^ Clinicians usually select different acupuncture treatments or combinations of multiple acupuncture therapies based on individual's condition, and the treatments have not yet been standardized. Hence, it is still necessary to find out what acupuncture treatment strategy should be preferred in clinical practice of dysphagia after stroke.

There are numerous acupuncture and related therapies for dysphagia after stroke and their efficacy has been evaluated by previous RCTs. However, the research quality is uneven, and the intervention mode is relatively simple compared with the clinical practice, without considering multiple factors together. NMA, recognized as a valuable tool, can integrate direct and indirect comparisons across a set of multiple interventions.^[30,31]^ Thus, we employed a NMA of relevant RCTs of acupuncture and related therapies approaches for dysphagia after stroke, including TA, EA, MA, SA, and do on, to evaluate the efficacy of different acupuncture treatments. As we have seen, this study will be the first NMA in this respect. The aim is to provide more objective and comprehensive evidence support and optimized plan for acupuncture therapeutic options for patients with dysphagia after stroke.

However, there are some potential limitations that are predictable in this NMA. Acupuncture therapy is widely used for stroke patients in China and other oriental countries, such as South Korea and Japan.^[32]^ However, we search only the English and Chinese literature, then the medical databases in languages such as Japanese and Korean will be excluded, which may lead to selection bias. In addition, this study will not focus on the difference of acupuncture techniques and acupoint selections, and the different RCTs intervention times cannot be absolutely unified. Although the inconsistency will be reduced by subgroup analysis, the results still need to be treated with caution.

Nevertheless, the ranking of different acupuncture therapies provided by this study is valuable, which can guide clinical decision makers to select the best treatment strategies for dysphagia after stroke. The findings and results of this study will be published in a peer-reviewed journal.

## Author contributions

Fanjie Xiong and Kai Song made the same contribution to the research and design, and wrote the original draft of the protocol. Fanjie Xiong has developed a search strategy. Fanjie Xiong, Kai Song and Ailing Huang will conduct literature retrieval and collation. Fanjie Xiong, Kai Song and Hong Zhang will evaluate the risk of bias in the literature. Data analysis and article writing will be done by Fanjie Xiong, Kai Song. Hong Zhang, as the corresponding author, will be responsible for overseeing every process of the audit review to control the quality of the study. All the authors have approved the publication of the protocol.
